# Advances in Crop Improvement and Delivery Research for Nutritional Quality and Health Benefits of Groundnut (*Arachis hypogaea* L.)

**DOI:** 10.3389/fpls.2020.00029

**Published:** 2020-02-21

**Authors:** Chris O. Ojiewo, Pasupuleti Janila, Pooja Bhatnagar-Mathur, Manish K. Pandey, Haile Desmae, Patrick Okori, James Mwololo, Hakeem Ajeigbe, Esther Njuguna-Mungai, Geoffrey Muricho, Essegbemon Akpo, Wanjiku N. Gichohi-Wainaina, Murali T. Variath, Thankappan Radhakrishnan, Kantilal L. Dobariya, Sandip Kumar Bera, Arulthambi Luke Rathnakumar, Narayana Manivannan, Ragur Pandu Vasanthi, Mallela Venkata Nagesh Kumar, Rajeev K. Varshney

**Affiliations:** ^1^ Research Program – Genetic Gains, International Crops Research Institute for the Semi-Arid Tropics (ICRISAT), Nairobi, Kenya; ^2^ Research Program – Genetic Gains, International Crops Research Institute for the Semi-Arid Tropics (ICRISAT), Hyderabad, India; ^3^ Research Program – West and Central Africa, International Crops Research Institute for the Semi-Arid Tropics (ICRISAT), Bamako, Mali; ^4^ Research Program – Eastern and Southern Africa, International Crops Research Institute for the Semi-Arid Tropics (ICRISAT), Lilongwe, Malawi; ^5^ Research Program – West and Central Africa, International Crops Research Institute for the Semi-Arid Tropics (ICRISAT), Kano, Nigeria; ^6^ Indian Council of Agricultural Research - Directorate of Groundnut Research (ICAR-DGR), Junagadh, India; ^7^ Main Oilseeds Research Station, Junagadh Agricultural University (JAU), Junagadh, India; ^8^ National Pulses Research Center, Tamil Nadu Agricultural University (TNAU), Pudukkottai, India; ^9^ Regional Agricultural Research Station, Acharya NG Ranga Agricultural University (ANGRAU), Tirupati, India; ^10^ Department of Genetics and Plant Breeding, Professor Jayashankar Telangana State Agricultural University (PJTSAU), Hyderabad, India

**Keywords:** aflatoxin, allergens, *Arachis hypogaea*, crop improvement, groundnut, oleic acid, science of delivery

## Abstract

Groundnut is an important global food and oil crop that underpins agriculture-dependent livelihood strategies meeting food, nutrition, and income security. Aflatoxins, pose a major challenge to increased competitiveness of groundnut limiting access to lucrative markets and affecting populations that consume it. Other drivers of low competitiveness include allergens and limited shelf life occasioned by low oleic acid profile in the oil. Thus grain off-takers such as consumers, domestic, and export markets as well as processors need solutions to increase profitability of the grain. There are some technological solutions to these challenges and this review paper highlights advances in crop improvement to enhance groundnut grain quality and nutrient profile for food, nutrition, and economic benefits. Significant advances have been made in setting the stage for marker-assisted allele pyramiding for different aflatoxin resistance mechanisms—*in vitro* seed colonization, pre-harvest aflatoxin contamination, and aflatoxin production—which, together with pre- and post-harvest management practices, will go a long way in mitigating the aflatoxin menace. A breakthrough in aflatoxin control is in sight with overexpression of antifungal plant defensins, and through host-induced gene silencing in the aflatoxin biosynthetic pathway. Similarly, genomic and biochemical approaches to allergen control are in good progress, with the identification of homologs of the allergen encoding genes and development of monoclonal antibody based ELISA protocol to screen for and quantify major allergens. Double mutation of the allotetraploid homeologous genes, *FAD2A* and *FAD2B*, has shown potential for achieving >75% oleic acid as demonstrated among introgression lines. Significant advances have been made in seed systems research to bridge the gap between trait discovery, deployment, and delivery through innovative partnerships and action learning.

## Introduction

Groundnut is an invaluable source of protein, calories, essential fatty acids, vitamins, and minerals for human nutrition (Willett et al., 2019). Groundnut consumption is reported to be associated with several health benefits (Kris-Etherton et al., 2008; Sabate et al., 2010; Guasch-Ferré et al., 2017). Indeed, a recent Lancet-commissioned publication concludes that transformation to healthy diets by 2050 requires substantial dietary shifts, including more than 100% increment in consumption of healthy foods, such as nuts, fruits, vegetables, and legumes (Willett et al., 2019). Higher consumption of total and specific types of nuts was found inversely associated with total cardiovascular disease and coronary heart disease (Guasch-Ferré et al., 2017). Qualified health claim linking early groundnut introduction and reduced risk of developing groundnut allergy was acknowledged by Food and Drug Administration (FDA) (FDA, 2017).

Groundnut is a rich source of dietary protein with ability to meet up to 46% of recommended daily allowance; essential vitamins especially E, energy from its oils and fats, and dietary fiber. It is also a rich source of minerals such as K, Na, Ca, Mn, Fe, and Zn among others and a rich source of biologically active compounds (arginine, resveratrol, phytosterols, and flavonoids). Zinc in particular, is one of the limiting micronutrients especially among rural households in Africa affecting especially infants and young persons (Wessells and Brown, 2012). This explains why groundnut is a rich base in therapeutic foods. The World Health Organization of the United Nations encourages consumption of groundnut-based “ready-to-use therapeutic foods” (RUTF) for community-based treatment of severe malnutrition. For example Plumpy'Nut^®^, used to treat severe acute malnutrition in children is i) calorie-dense, high in proteins, vitamins, and minerals; ii) simple to deliver and administer without training; iii) fast acting; iv) affordable; v) culturally acceptable; vi) packed in single-serve packets; vii) requires little preparation before use; viii) equipped with adequate shelf-life and stability; ix) storable in varied climatic conditions and temperature; x) resistant to bacterial contamination; and xi) not causative of addiction in children (Ojiewo et al., 2015).

In Ghana, groundnut has been identified as a nutrient-dense food with high capacity to deliver nutrition and income outcomes to producers and consumers. The crop scored highly for nutrition quality, affordability, acceptability, integrity, and business/investment interest (Anim-Somuah et al., 2013). In Malawi and Tanzania, groundnut is prioritized as a crop for diversifying the economy, being included in the national investment strategy and national agricultural development blue print (Anonymous, 2014). In spite of being a cheap source of dietary benefits per-capita consumption is still low even among major producers such as Malawi and Tanzania. For example, our evaluation studies show that per-capita consumption in Mtwara, Southern Tanzania, a major groundnut producer is low. From a sample of 224 farmers in Mtwara, only 3% of mothers reported to feed their children (6–23 months old) food containing groundnut, yet animal protein consumption in the same age-group of children was only less than 1%. From the same study, similar trends were observed in consumption patterns among women of reproductive age where groundnut could serve as a vital source of iron and zinc. In Malawi per capita consumption is 8 kg, doubling from 4 kg in the mid-2000s after intervention by International Crops Research Institute for the Semi-Arid Tropics (ICRISAT). In fact groundnut production and consumption in Malawi, is relatively higher than neighboring countries, with 47.9% of households consuming groundnut food products more than four times a week (Seetha et al., 2018a).

In another study in Malawi among respondents of diverse genders and regions, greater than 70% reported to consume groundnuts in different forms at least three times a week (Gama et al., 2018). Data from Nigeria also indicates the highest per capita consumption of top 20 groundnut consumers surveyed (International Nut and Dried Fruit, 2018). These studies however, indicate consumption either among a mixed group of adults or the general population with no stratification of consumption among nutritionally vulnerable groups such as infants, young children, and women of reproductive age. Promotion of consumption among these groups is still warranted and vital as they bear the burden of iron and zinc deficiency, minerals that are rich in groundnuts.

If groundnut consumption is to be increased in such areas where micronutrient deficiencies remain of public health importance, crop improvement must address productivity and nutritional quality challenges. Thus, concerted efforts are needed to develop high yielding, nutrient dense, and market preferred groundnut varieties.

Equally important is the need to improve functionality of groundnut seed systems to improve access and adoption of improved varieties. Effective and efficient seed systems (seed value chains), support delivery, and access to improved crop varieties on time and at affordable price. It also supports planning demand and supply from the farm to national levels, a critical point for seed security (Sperling, 2008; McGuire and Sperling, 2016). Seed is the vehicle that delivers all the millions of base pairs of DNA that pattern into the genome of a plant expressed in its phenome. Upstream research should therefore work with delivery in mind and form strategic partnerships that create real impacts on the ground besides high impact publications^1^ ^,^ ^2^. In this review, we have highlighted some of the advances in crop improvement efforts related to nutritional and oil quality as well as addressing key challenges of groundnut grain nutrient quality, with a focus on aflatoxin contamination and allergens. We also highlight efforts to link upstream research and delivery of nutritionally superior groundnut varieties for food, nutrition, and income security.

## Methods, Limitations, and Bias

This synthesis paper is highlighting major efforts, achievements, lessons learned, challenges, and gaps in the process of development to delivery of nutrient dense and health safe groundnut. For the most of the work around development of low aflatoxin, low allergen, and high oleic acid groundnut, emphasis is on the work done by the ICRISAT together with its network of national and international partners. A significant portion of literature cited and reported data is published work stemming from major projects hosted by ICRISAT at various times.

ICRISAT hosted a large project on “Tropical Legumes: Improving Livelihoods for Smallholder Farmers: Enhanced Grain Legume Productivity and Production in Sub-Saharan Africa and South Asia between 2007–2010 (Phase 1), 2011–2014 (Phase 2) and 2015–2019” (TLIII). The Tropical Legumes projects put emphasis on developing, testing, and promoting improved crop cultivars to enhance legume productivity and production in the drought-prone areas of target regions and countries. Besides other emphasis, the partners put concerted efforts in developing cultivars tolerant to drought and the major production and consumption constraints including aflatoxin challenge, groundnut allergens, and lipid profile using concerted approaches such as marker-assisted selection and genetic engineering. ICRISAT also hosted the Consultative Group on International Agricultural Research (CGIAR) Research Program on Grain Legumes (CRP-GL) which progressed into CGIAR Research Program on Grain Legumes and Dryland Cereals (CRP-GLDC) with work packages covering crop improvement of groundnut. Results from these projects together with some of their precursors and successors form the bulk of literature cited here, thereby explaining the bias toward ICRISAT. This synthesis paper includes limited literature on the major groundnut research too as examples that can be referred to in the process of mainstreaming orphaned crops.

## Advances in Crop Improvement to Mitigate Groundnut Aflatoxin Contamination

Aflatoxin is a dangerous mycotoxin produced by the fungus *Aspergillus flavus* Link : Fr, from which it draws its name. Aflatoxin contamination, is particularly common in all starchy agricultural food products, because of the ubiquitous nature of the *Aspergillus* species, a saprophyte that starts infecting crop products, especially grain, from the field to storage in the process producing aflatoxins (Njoroge et al., 2017; Seetha, et al., 2018b); aflatoxin laced food products certainly affect nutrition benefits and trade.

Studies suggest that the aflatoxin is carcinogenic, immunosuppressive (reduction of the activation or efficacy of the immune system), hepatotoxic (liver toxicity), and teratogenic (abnormalities of physiological development) in nature and thus has adverse impacts on human and animal health thus affecting nutrition and trade in many African and Asian countries (Amaike and Keller, 2011; Kensler et al., 2011; Monyo et al., 2012; Kamika et al., 2014; Mupunga et al., 2014; Njoroge et al., 2016; Njoroge et al., 2017; Agbetiameh et al., 2018; Norlia et al., 2018; Lien et al., 2019). Exposure to aflatoxins, particularly aflatoxin B1 (AfB1), is associated with increased risk of developing cirrhosis and liver cancer (Chu et al., 2017). Africa in particular, children are exposed to aflatoxin contamination *in utero* and throughout the weaning period and beyond (Turner et al., 2007; Khlangwiset et al., 2011; Watson et al., 2018; Seetha et al., 2018b). In a recent survey in Northern Nigeria (Ajeigbe et al., 2018), AfB1 concentrations in kuli kuli, a groundnut product widely consumed in different forms by a vast majority of Nigerians, range between 4.10 and 268.00 μg/kg. Indeed, 87–100% of kuli kuli consumed in Nigeria is contaminated by aflatoxin. The situation of several other groundnut-based products are not very different from that of kuli kuli. For example, between 91 and 96% of roasted groundnut sold at different locations across Nigeria are contaminated by AfB1 with concentrations ranging between 1 and 65 μg/kg.

Mitigating exposure to aflatoxins positively impacts growth of children (Seetha et al., 2018b). Given that groundnut is a common weaning food in many rural farming households, reducing exposure to aflatoxins will improve achievement of the Sustainable Development Goals (SDG) two (ending hunger and all forms of malnutrition by 2030), the WHO goal of reducing stunting of children under 5 years by 40%. Given the evidence showing an association between aflatoxin exposure and stunting, aflatoxin contamination of nutrient dense crops such as groundnut needs to be addressed to break the vicious links to aflatoxin contamination. Additionally, epidemiological studies have demonstrated a strong link between (AfB1) consumption and cancer occurrence as well as liver toxicity further compounding the evidence on the negative health effects of aflatoxin contamination.

A recent study conducted in Democratic Republic of Congo indicated that awareness of consumers on the dangers and mitigation measures of aflatoxin contamination in groundnut is still limited (Udomkun et al., 2018). Similarly, in a study conducted in 2018 by ICRISAT, 80.7 and 70% of households surveyed indicated that they had seen green moldy grain in Tanzania and Malawi, respectively. However, only 3.3% in Tanzania and 50% in Malawi had heard about aflatoxin contamination. The higher level of aflatoxin awareness in Malawi is mainly due to concerted efforts of the National Smallholder Farmers' Association of Malawi (NASFAM) backstopped by ICRISAT-Malawi and national groundnut program researchers (Ojiewo et al., 2018a). Generally, there seems to be a very strong disconnect from the efforts that upstream researchers are making on aflatoxin control from the intended users of research outputs. The same could be said of other aspects, not only of groundnut crop improvement research, but of many other crops. Nevertheless, as shown in this article, ICRISAT together with its partners are doing their best to bridge this disconnect and has already achieved success by integrating genomics in breeding to develop disease resistant (Varshney et al., 2014), high oleate (Janila et al., 2016; Bera et al., 2018), and low allergen lines (Pandey et al., 2019c) among others.

The use of host-plant resistance to *A. flavus* offers cost-effective and environmentally sound management strategy for mitigation of the aflatoxin threat in groundnut. Aflatoxins have zero phytotoxicity but high mammalian toxicity hence the absence of any mitigation metabolism to mitigate its production *in planta*. There are three major mechanisms that have been identified that reduce infection of grain: *in vitro* seed colonization (IVSC), reduced pre-harvest aflatoxin contamination (PAC), and reduced aflatoxin production (AP). These resistances can be broadly classified as pod infection (pod wall), seed invasion and colonization (seed coat), and aflatoxin production (cotyledons). While the resistance to pod infection is attributed to physical barriers due to the pod-shell structure, seed invasion and colonization is correlated with density and thickness of palisade cell layers, presence of fungistatic phenolic compounds, wax layers, and absence of microscopic fissures and cavities. These resistance components are highly variable, independent, appearing to be governed by different genes with no significant relationships within, and have been breeding focuses to identify resistant genotypes (Nigam et al., 2009). Stable resistance can be achieved by accumulating favorable alleles for IVSC, PAC, and AP, in addition to deployment of pre- and post-harvest management practices (Pandey et al., 2019a). Studies to identify low groundnut genotypes that experience aflatoxin contamination material, has been conducted by ICRISAT and partners for several years using genebank and other germplasm, with slow and limited progress made. Notwithstanding, it has led to identification of material such as ICGV 88145, ICGV 89104, ICGV 91278, ICGV 91283, ICGV 91284 (Nigam et al., 2009) and 73‐33, ICGV 89063, ICGV 89112, J11 and 55‐437 (Mayeux et al., 2003), and ICG 23. J 11 and 55‐437, released in West Africa are known to accumulate low pre-harvest levels of aflatoxin (Mayeux et al., 2003). However, no aflatoxin resistant varieties have been released yet (Arias et al., 2018; Desmae et al., 2019).

ICRISAT's groundnut breeding programs in Malawi, Mali, and India have initiated breeding of low aflatoxin contaminated groundnut, developing populations and screening lines. At ICRISAT-Malawi, development of populations using eight popular varieties in the region and three sources of aflatoxin resistance started in 2012. The eight and three parental lines included: CG 7, Pendo, ICGV-SM 90704, JL 24, ICGV-SM 01721, ICGV-SM 01711, ICGV-SM 99557, ICGV-SM 99555; and J11, ICGV 95494, Ah 7223 respectively. A second set using a ICG 23, ICG 6402, and ICG 1122 as donor male parents for low aflatoxin contamination has been developed and are at F7 stage from which 32 lines were selected in 2018 to constitute a new population using a three way cross (**Table 1**). These new sources are additionally tolerant to drought and early maturing.

**Table 1:** Populations developed involving eight female high aflatoxin contamination genotypes and three low aflatoxin male genotypes to introgress low aflatoxin accumulation in the elite eight material.

S no.	Female	Positive traits	Male	Positive traits	Cross
1	CG7	High oil content (48–50%), high yield (> 2,500 kg/ha), medium duration (120–130 days)	J11	Low aflatoxin	CG7 × J11
			ICGV 95494	Low aflatoxin	CG7 × ICGV 95494
			Ah 7223	Low aflatoxin	CG7 × Ah 7223
2	Pendo	Short duration 90–100 days, medium seeded and good for confectionery	J11	Low aflatoxin	Pendo × J11
			ICGV 95494	Low aflatoxin	Pendo × ICGV 95494
			Ah 7223	Low aflatoxin	Pendo × Ah 7223
3	ICGV-SM 90704	Rosette resistant, high yielding (> 2,000 kg/ha), medium duration; low oil content and good for relish	J11	Low aflatoxin	ICGV-SM 90704 × J11
			ICGV 95494	Low aflatoxin	ICGV-SM 90704 × ICGV 95494
			Ah 7223	Low aflatoxin	ICGV-SM 90704 × Ah 7223
4	ICGV-SM 01721	Large seeded, tolerant to rosette, high yield (> 2,500), medium duration (120–130 days)	J11	Low aflatoxin	ICGV-SM 01721× J11
			ICGV 95494	Low aflatoxin	ICGV-SM 01721× ICGV 95494
			Ah 7223	Low aflatoxin	ICGV-SM 01721× Ah 7223
5	JL24	Short duration (extra early—90 days), good taste, ease of blanching (confectionery)	J11	Low aflatoxin	JL24 × J11
			ICGV 95494	Low aflatoxin	JL24 × ICGV 95494
			Ah 7223	Low aflatoxin	JL24 × Ah 7223
6	ICGV-SM 01711	Large seeded, high yielding (> 2,500 kg/ha), resistant to groundnut rosette disease (GRD), medium duration (120–130 days)	J11	Low aflatoxin	ICGV-SM 01711 × J11
			ICGV 95494	Low aflatoxin	ICGV-SM 01711 × ICGV 95494
			Ah 7223	Low aflatoxin	ICGV-SM 01711 × Ah 7223
7	ICGV-SM 99557	Short duration (100–110 days), good for confectionery; resistant to GRD	J11	Low aflatoxin	ICGV-SM 99557× J11
			ICGV 95494	Low aflatoxin	ICGV-SM 99557 × ICGV 95494
			Ah 7223	Low aflatoxin	ICGV-SM 99557× Ah 7223
8	ICGV-SM 99555	Short duration (100–110 days), good for confectionery, resistant to GRD	J11	Low aflatoxin	ICGV-SM 99555 × J11
			ICGV 95494	Low aflatoxin	ICGV-SM 99555 × ICGV 95494
			Ah 7223	Low aflatoxin	ICGV-SM 99555 × Ah 7223

Similarly, in ICRISAT-Mali, screening of ICRISAT's groundnut mini core accessions was conducted between 2008 and 2013 resulting in identification of low aflatoxin contamination sources especially due to pre-harvest aflatoxin contamination. The accessions are ICG 13603, ICG 1415, ICG 14630, ICG 3584, ICG 5195, ICG 6703, and ICG 6888 (Waliyar et al., 2016). Some of these materials have been used in population generation with, more than 130 populations developed between 2015 and 2018. In ICRISAT-India, multi-parent advanced generation inter-cross (MAGIC) populations have been developed by crossing eight genotypes possessing low contamination conditioned by at least one of the three mechanisms (Pandey et al., 2019a). These parents include: ICGV 88145, ICGV 89104, U4-7-5, VRR 245, ICG 51, ICGV 12014, ICGV 91278, and 55-437. The MAGIC lines have been phenotyped for two seasons for PAC and AP in addition to genotyping with high-density 58K Axiom_Arachis single nucleotide polymorphism (SNP) array (Pandey et al., 2017a). Further genetic studies using association mapping and further characterization of highly resistant lines is in progress.

Advances in genomics provide unprecedented opportunity for improving resistance to *A. flavus* infection and its associated aflatoxin contamination. Such genomic tools, provides opportunity to address the high genotype by environment interaction during trial evaluations that has slowed genetic gain (Nigam et al., 2009). Genomic advances include sequencing of groundnut diploid progenitors (Bertioli et al, 2016; Chen et al., 2016; Lu et al., 2018) and the cultivated tetraploid groundnut (Bertioli et al., 2019; Chen et al., 2019; Zhuang et al., 2019); that provides a scaffold for decoding genetics of host resistance to *A. flavus* pathogenesis and its associated aflatoxin metabolite production. Additionally, advances in biotechnology, especially recombinant DNA in groundnut by overexpressing antifungal plant defensins MsDef1 and MtDef4.2 against *A. flavus* pathogenesis, and through host‐induced gene silencing (HIGS) of aflM and aflP genes (Sharma et al., 2018) from the aflatoxin biosynthetic pathway (see next section for details) provide promise of deployment of plant and pathogen derived defense systems to reduce and or eliminate aflatoxin contamination in groundnut.

### Aflatoxin Mitigation in Groundnut Using Host‐Induced Gene Silencing and Transgenic Approaches: Technology and Translation

**Figure 1:**
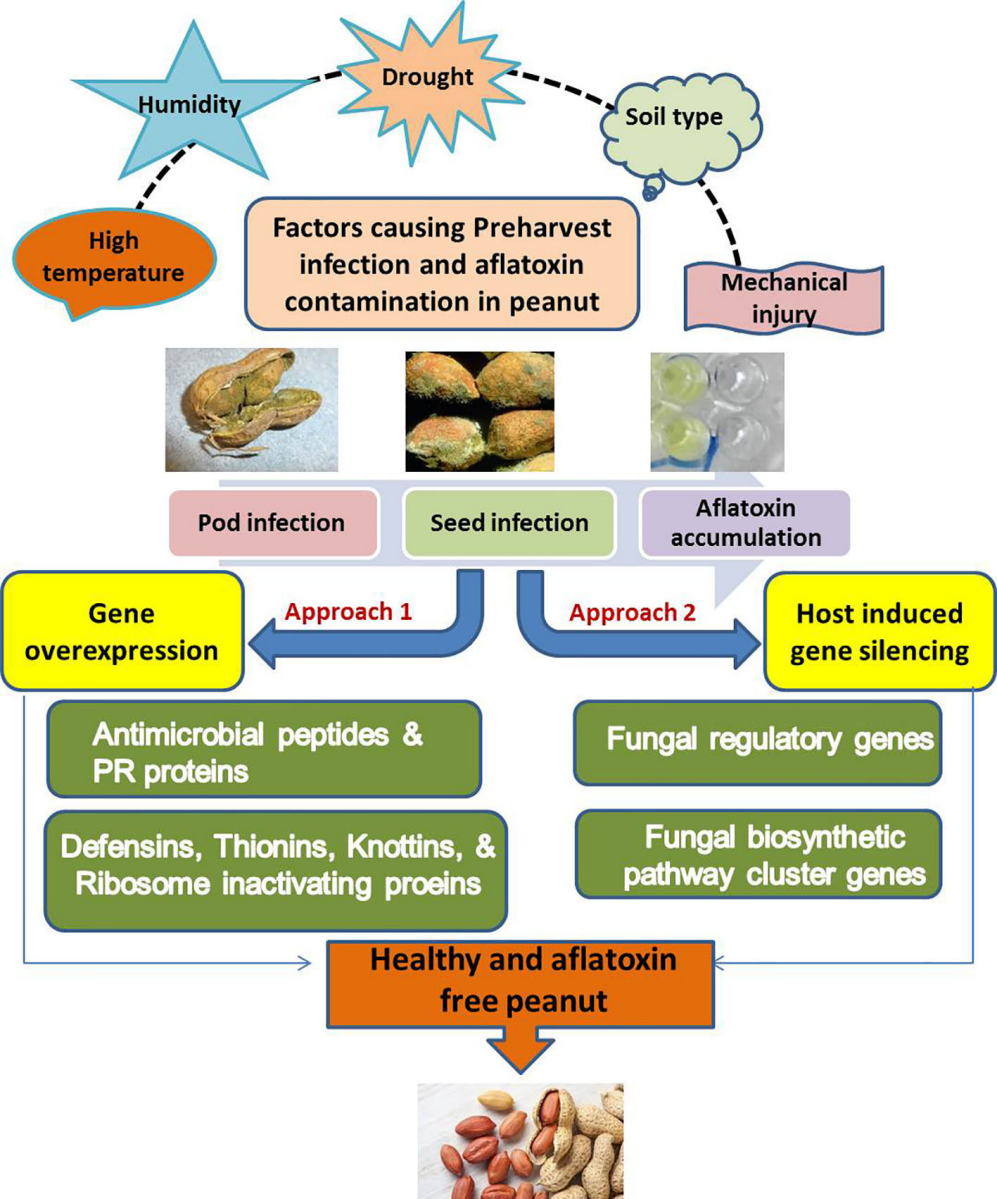
Schematic representation of an integrated biotechnological approach for pre-harvest aflatoxin management.

Given the fact that aflatoxins are not phytotoxic, it is improbable that a host-pathogen co-evolution exists that can generate natural mechanisms for resistance. However, infection by *A. flavus*/*Aspergillus parasiticus* poses a threat to seed and its precious embryo, and therefore, a threat to a plant's transmission of the gene to the next generation. Thus a focus on exploiting pathogen/host interactions that minimizes pre-harvest infection has received renewed efforts. A second alternative is to focus on genetic engineering of pathogenicity genes that interfere with aflatoxin metabolism in the fungus. Deployment of such genes requires elucidation of aflatoxin metabolism in *Aspergillus*. The progress though, has been considerably slow, due to limited understanding of the resistance mechanism and associated markers (Luo et al., 2009). The use of “competitive atoxigenic” fungal technology (CAFT), deploying promiscuous atoxigenic *Aspergillus* strains has been fairly successful in reducing levels of aflatoxin contamination in maize (Atehnkeng et al., 2014), but the same may not be applicable in groundnut, a subterranean legume where mold growth on the grain reduces quality. These scenario necessitated the deployment of genetic engineering approach to develop transgenic resistance at ICRISAT, where a two-pronged strategy was used. High levels of immunity to *A. flavus* infection and colonization was achieved by overexpression (OE) of antifungal plant defensins from alfalfa, and through HIGS of the aflatoxin metabolism (**Figure 1**).

By expressing double stranded RNA molecules of *Aspergillus* in the groundnut–host system, the fungal toxin production pathway was interrupted, making the fungus incapable of aflatoxin production and accumulation (Bhatnagar-Mathur et al., 2015). The chimeric genes were designed for localized to extracellular spaces and endoplasmic reticulum. The *OE-Def* events showed higher expression of defensins at different pod development stages and maintained steady transcript abundance (up to 70-fold) of the respective defensin until 72 h post inoculation (hpi), compounding resistance to fungal growth. Further *OE-Def* events showed reduced conidiophore length and conidial head width and had very low fungal load compared to the wild-type control. Similarly, HIGS vectors carried cauliflower mosaic virus (CaMV) 35S promoter-regulated hp-RNA (hairpin-RNA) cassettes comprising of synthetic DNA incorporating sections of aflP/omtA and aflM/ver-1 genes cloned as inverted repeats around the PR10 intron and used for transformation (Bhatnagar-Mathur et al., 2015; Sharma et al., 2018).

Molecular analysis confirmed gene integration and expression in the events and aflatoxin B1 was estimated using HPLC (high-performance liquid chromatography) analysis. Significant reduction in transcription of early, middle, and late pathway genes were observed in both infected OE-Def and HIGS lines. OE-Def and HIGS lines maintained the reactive oxygen species (ROS) homoeostasis, a critical pathogenesis mechanism (Liu et al., 2010; Jun and Zhulong, 2015). This was possibly through positive regulation of the transcription of *SOD* and *CAT* genes. Several events accumulated <4 ppb AfB_1_ compared to >2,000 ppb detected in controls indicating very high levels of resistance to aflatoxin contamination. Significant reductions in transcription of early, middle, and late pathway genes were observed in infected OE-Defensin and HIGS lines.

Progeny from six promising transformation events assayed for A. flavus infection and subsequent aflatoxin content, revealed high levels of consistency, exhibiting trait stability across successive generations. In fact, the stable defensin and HIGS transformation events exhibited large aflatoxin contamination reduction, accumulating 0.5–4 ppb of AfB_1_ compared to >2,000 ppb in wild type (Bhatnagar-Mathur et al., 2015; Sharma et al., 2018). Experiments are underway to introgress these “traits” into elite backgrounds. Preliminary fungal bioassays with F_2_ seeds derived from eight cross combinations, also demonstrated very low levels of aflatoxin (< 10 ppb) compared to wild type counterparts providing reasonable confidence to initiate deployment in groundnut breeding pipelines.

Furthermore, new set of HIGS lines carrying 4 hp-RNAs are currently under development to silence multiple genes in *A. flavus* by generating multi-target RNA interference (RNAi) signals for genes involved in transcriptional regulation of genes required for developmental processes of sclerotium morphogenesis and conidiation in *A. flavus*, in addition to the ones that regulate aflatoxin production. Preliminary results with groundnut seed carrying multi-target RNAi signal in T_1_ and T_2_ generation showed significant decrease in fungal colonization and aflatoxin production. This shows that down-regulation of genes vital for fungal growth and aflatoxin production through RNAi would be effective in enhancing aflatoxin resistance in groundnut plants.

## High Oleic Groundnut Varieties for Food and Nutrition Security

High oleic trait is an important quality parameter, which determines the flavor, stability, shelf-life, and nutritional quality of groundnut and groundnut products. High oleic groundnut is preferred by food processing and edible oil industry for its extended shelf life and high quality respectively. Groundnut oil and processed food products made using high oleic grain have 10-fold enhanced shelf life compared to regular groundnut (O'Keefe et al., 1993; Braddock et al., 1995). Oxidative rancidity is common in oils with high levels of polyunsaturated fatty acid due to the presence of double carbon bonds that degrade over time producing acids, aldehydes, ketones, and hydrocarbons (Moore and Knauft, 1989). Increased consumption of high-oleate groundnuts as compared to diets without groundnuts at all also has been shown to be linked to improved cardiac health (Guasch-Ferré et al., 2017). Replacement of other frying oils with a high content of polyunsaturated fatty acids will lead to better stability of frying oils, but without the negative cardiac health impacts of trans-fatty acids from hydrogenated oils or peroxide, polyaromatic hydrocarbon formation in polyunsaturated fatty acid (PUFA) oils during prolonged heating, thereby replacing less healthy alternative frying oils (Kratz et al., 2002).

Two fatty acids—namely oleic acid (monounsaturated fatty acid, MUFA) and linoleic acid (PUFA) accounts for up to 80% of the groundnut oil. The remaining fatty acids including palmitic, stearic, arachidic, gadoleic, behenic, and lignoceric acids constitute 20% with palmitic acid a saturated fatty acid alone contributing 10% (Kavera et al., 2014). High oleic groundnut varieties have a mutated form of the fatty acid dehydrogenase (FAD) gene. This gene encodes an enzyme delta-12-desaturase (oleoyl-PC desaturase) which catalyses addition of a second double bond onto oleic acid to produce linoleic acid. If the enzyme is inactivated, then oleic acid accumulates in the oil bodies resulting in oleic acid contents of more than 80%, while the linoleic acid content remains around 2–5%. Due to the allotetraploid nature of groundnut there are two homeologous gene sequences (FAD2A and FAD2B) believed to originate from the two progenitor species genomes—Arachis *duranensis* and Arachis *ipaensis* (Bertioli et al., 2019; Chen et al., 2019; Zhuang et al., 2019). Mutations in either one of these genes leads to small increase in oleic acid content of by over 60% (Nawade et al., 2016). However, the presence of both the mutant alleles of *FAD2A* and *FAD2B* genes is essential for achieving >75% oleic acid (Pandey et al., 2014) which have clearly been observed among introgression line developed using allele-specific markers (Janila et al., 2016).

Norden et al. (1987) identified the first natural high-oleate groundnut mutant line, F435 with about 80% oleic acid and 2% linoleic acid. The first high oleate groundnut variety, SunOleic 95R was bred in USA through conventional breeding (Gorbet and Knauft, 1997). Following the identification of linked allele-specific (Chen et al., 2010), and cleaved amplified polymorphic sequence markers for both the ahFAD2 genes (ahFAD2A and ahFAD2B; Chu et al., 2009), marker assisted backcross breeding (MABC) and marker assisted selection (MAS) were used to improve oleic acid content of a nematode resistant variety, ‘Tifguard' in USA (Chu et al., 2011). Techniques such as HybProbe SNP assay (Bernard et al., 1998) and multiplex real-time PCR assay (Barkley et al., 2010) were also utilized in selecting the heterozygous and homozygous breeding lines for both mutant alleles. Recently, high-oleic lines have been developed using MAS and MABC in Spanish and Virginia Bunch varieties in India (Janila et al., 2016; Bera et al., 2018). The use of markers in breeding considerably reduced the time and population size in different backcross generations. High oleic groundnuts have also been developed and released for cultivation in Australia, Brazil, Argentina, and China. Interestingly, Australia is the only country cultivating 100% high oleic groundnut. Evaluation of high oleate lines is under testing in many countries in Africa and Asia. The high oleic acid in cooking oil decreases the risk of cardiovascular disease (CVD) by reducing the levels of serum low-density lipoprotein (LDL) cholesterol and maintaining the levels of high-density lipoproteins (HDL) (Rizzo et al., 1986); as compared to oils with high proportion of saturated fatty acids.

At ICRISAT, looking at the potential food industry needs and consumer health benefits, the high oleic breeding and testing pipelines are furthered by breeding high oleic in the background of elite/popular adapted varieties for different agro-ecologies as well as pyramiding multiple traits into a single cultivar. The ongoing groundnut breeding program incorporates the high oleic trait into biotic and abiotic stress resistant/tolerant cultivars, and different growth habits. In terms of biotic stresses such as late leaf spot and rust, although resistant lines were developed in the past using conventional breeding methodologies, majority of these resistant lines have long maturity. In this context, molecular markers were identified associated with resistance to these foliar diseases (Khedikar et al., 2010; Sujay et al., 2012; Pandey et al., 2017b). Subsequently, by using molecular markers and MABC approaches, the first set of foliar disease resistant lines were developed in three genetic backgrounds (Varshney et al., 2014). At present, late leaf spot (LLS) and rust resistance traits are being combined with the high oleic acid trait in the breeding programs both at ICRISAT and several national agricultural research systems (NARS) in Asia and Africa. The pyramided lines for foliar disease resistance and high oleic acid trait are currently under different stages of evaluation. The generation interval is reduced by using glass house facilities for generation advancement, and deploying genotyping, rapid and non-destructive phenotyping using near-infrared reflectance spectroscopy (NIRS), and early generation testing in target locations resulted in enhanced rate of genetic gain in high oleic breeding pipeline. SNPs for FAD2B mutant allele in F2 generation and phenotyping of harvested kernels from F2 single plants are used to make selection decisions in early generations.

**Figure 2:**
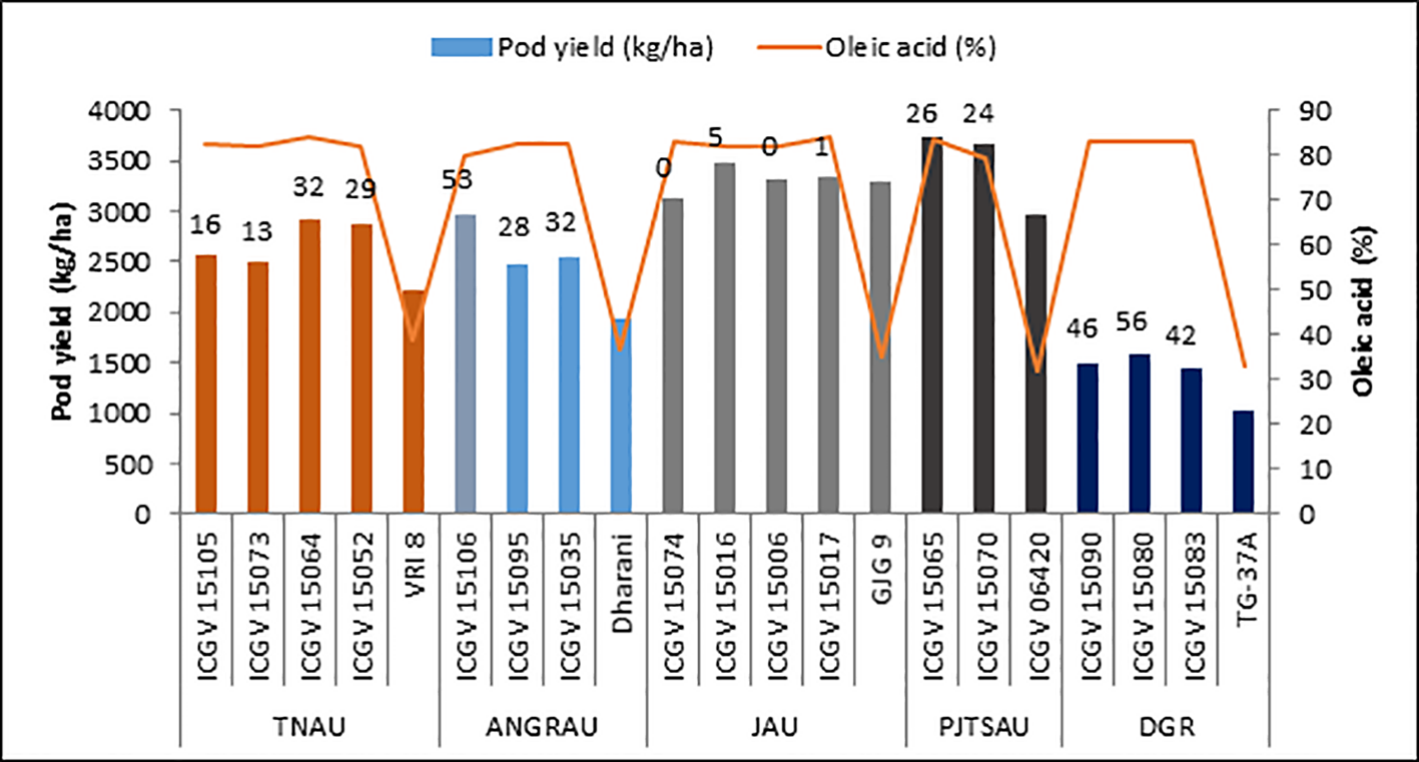
Performance of 16 high oleic lines under multi-location evaluation trials conducted during rainy season, 2016. These 16 lines were recommended for All India Co-ordinated Research Project on Groundnut (AICRP-G) testing based on their superior performance over the local check at respective location. Figures at the top of the bar indicates percentage increase in pod yield over the best local check. Oleic acid was measured by gas chromatography. TNAU, Tamil Nadu Agricultural University; ANGRAU, Acharya N G Ranga Agricultural University; JAU, Junagadh Agricultural University; PJTSAU, Prof Jayashankar Telangana State Agricultural University; DGR, ICAR-Directorate of Agricultural Research.

Bold seeded high oleic varieties with low oil content is preferred by the food processing industries. High amount of linoleic acid in the oil is not good for cooking purposes as it is vulnerable to oxidative rancidity and becomes thermodynamically unstable when heated at high temperature (Kratz et al., 2002). Oleic acid has 10-fold higher auto-oxidative stability than linoleic acid (O'Keefe et al., 1993) and therefore, with high oleic to linoleic acid ratio (O/L ratio), groundnut and its products have longer shelf life than normal lines (Bolton and Sanders, 2002). In high oleic lines the linoleic acid is reduced and oleic acid is increased. Keeping this in perspective two low oil containing bold seeded parents—ICGV 06110 and ICGV 07368 [70–80 g hundred seed weight (HSW)] and two high oil containing medium seeded parents—ICGV 06142 and ICGV 06420 (37–40 g HSW) were initially used as recurrent parents in a crossing program with SunOleic 95R being the donor parent for the high oleic trait. The first set of 64 high oleic lines developed at ICRISAT had a 100 seed mass of 30–40 g. These 64 lines were evaluated in multi-location trials conducted at five locations (Tamil Nadu Agricultural University, Coimbatore, Tamil Nadu; Acharya NG Ranga Agricultural Univ., Tirupathi, Andhra Pradesh; Junagadh Agricultural Univ., Junagadh, Gujarat; Prof. Jayashankar Telangana Agricultural Univ., Palem, Telangana; Indian Council of Agricultural Research-Directorate of Groundnut Research, Junagadh, Gujarat) representing Western, Central, and Sothern India and a subset of 16 high oleic lines were proposed for national evaluation under All India Co-ordinated Research Project on Groundnut (AICRP-G) (**Figure 2**).

**Figure 3:**
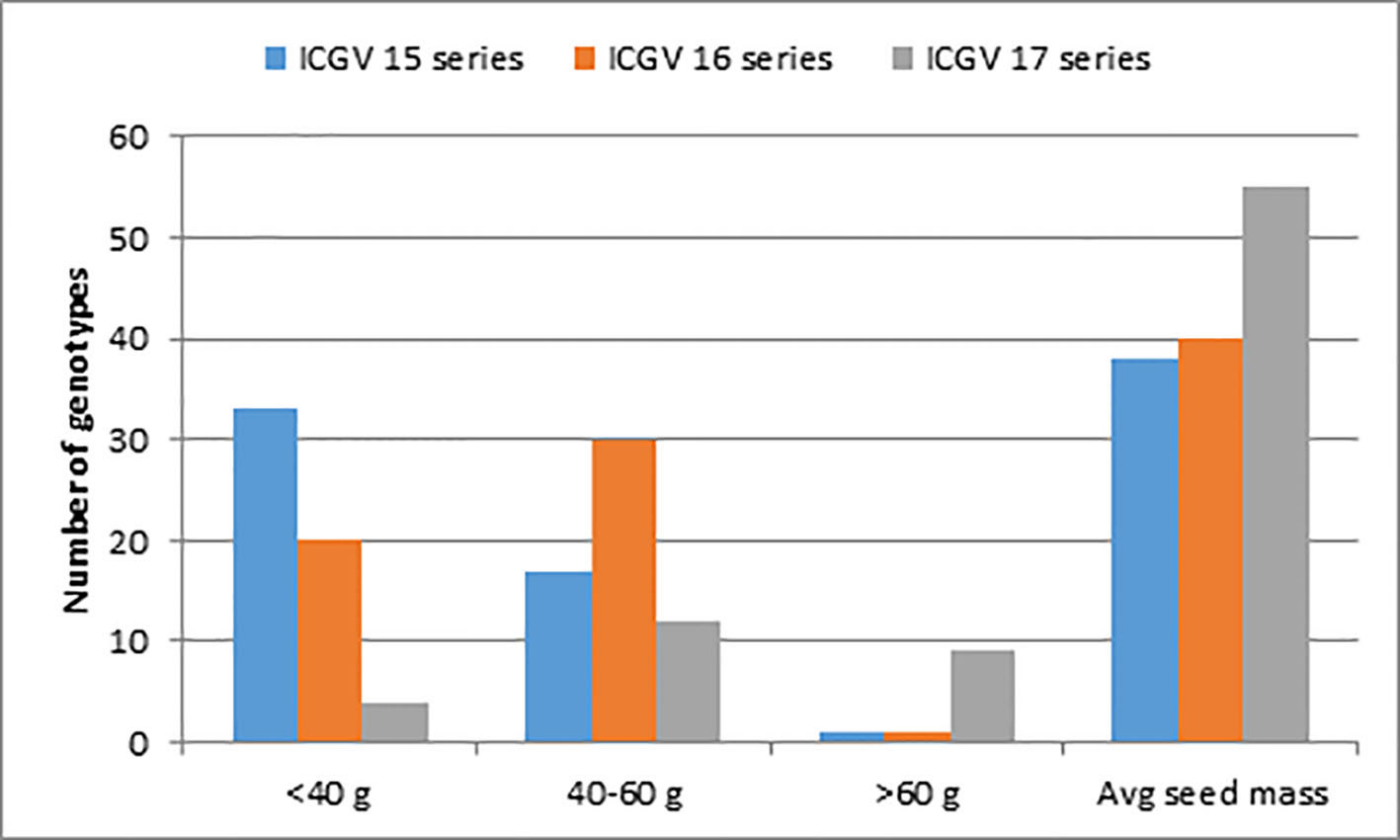
Progress in seed mass of high oleic lines evaluated at International Crops Research Institute for the Semi-Arid Tropics (ICRISAT) over three consecutive years. ICGV 15, 16, and 17 series indicate lines in the selection year 2015, 2016, and 2017, respectively.

The results from the study on global homogenous groundnut zones show that the similarity between African and Asian locations is much higher and hence need to choose collaboration partners across the globe as a way to achieve higher impact of investment (https://www.semanticscholar.org/paper/Global-homogenous-groundnut-zones-%E2%80%93-a-tool-to-the-Mausch-Bantilan/1d698be7f3dc50728fbc6784b532069c73ae85d0). Selection for the large kernel size in subsequent cycles and recycling of elite lines as parents, it was possible to achieve a significant yield gain in the 100-seed mass from an average of 38 to 55 g from 2015 to 2017 (**Figure 3**). Size distribution of the kernels that give the proportion of different size of kernels is another key criterion used in selection advancement decisions in high-oleic breeding pipelines.

Some of the high oleic lines developed at ICRISAT-India were evaluated in Nigeria (27 lines) and Mali (9 lines). Preliminary results show adaptability of some lines with relatively higher pod yield of up to 2.4 t/ha (**Table 2**). Many of the lines were tolerant to early leaf spot (*Cercospora arachidicola*) and late leaf spot (*Cercosporidium personatum*), with severity scores of 4 and 5. The lines displayed stay green character associated with tolerance to drought, with lines such as ICGV 15059, ICGV 15074, and ICGV 16001 having a haulm yield of more than 3.4 t/ha in Mali. Similarly, in Nigeria, lines ICGV 15060 (4.5 t/ha), ICGV 15065 (4.1 t/ha), and ICGV 15052 (4 t/ha) were the top three at BUK while ICGV 15034 (3.6 t/ha), ICGV 15064 (3.7 t/ha), and ICGV 15070 (4.6 t/ha) were the top three at Minjibir in haulms yield. Similar evaluations of 23 lines are under way in the East and Southern Africa breeding program, to identify best candidates for further evaluation and/or use in line conversions. In east and southern Africa two approaches are being used to develop high oleate groundnut. Firstly from the groundnut minicore (Upadhyaya et al., 2010) high oleate donor germplasm such as ICG 1274, ICG 5221, ICG 5475, ICG 6766, ICG 6646, ICG 6201 are being used to improve the oil quality of adapted and popular varieties in the region. The variety CG7 that has over 48% oil content for example is targeted to improve oil quality while, food and confectionary popular varieties such as ICGV-SM 90704 and ICGV-SM 08503 respectively, among others is have been target.

**Table 2:** Dry pod and haulm yield of high oleic lines in Nigeria (BUK and Minjibir) and Mali (Samanko) during 2018 main rainy season.

Variety	Dry pod yield (kg/ha)			Dry haulm yield (kg/ha)		
	BUK	Minjibir	Samanko	BUK	Minjibir	Samanko
ICGV 15023	1,075.0	1,250.0	1,104.2	1,733.3	2,558.3	3,093.8
ICGV 15059	1,258.3	1,050.0	520.8	2,366.7	933.3	3,416.7
ICGV 15070	1,300.0	1,650.0		3,091.7	4,641.7	
ICGV 15025	1,450.0	1,500.0	364.6	2,500.0	2,441.7	3,500.0
ICGV 16002	1,466.7	1,625.0		2,541.7	3,058.3	
ICGV 15090	1,533.3	1,383.3		2,875.0	1,675.0	
ICGV 15051	1,541.7	1,483.3		2,358.3	2,150.0	
ICGV 15033	1.550.0	1,525.0		2,575.0	2,033.3	
ICGV 16010	1.558.3	1,308.3	1,104.2	2,866.7	3,358.3	2,781.3
ICGV 15034	1,583.3	1,458.3		2,625.0	3,575.0	
ICGV 15080	1,608.3	1,675.0		2,466.7	3,083.3	
ICGV 15046	1,616.7	1,283.3	1,218.8	3,025.0	2,241.7	2,656.3
ICGV 15055	1,625.0	1,566.7		2,066.7	2,941.7	
ICGV 16001	1,633.3	1,641.7	1,468.8	3,091.7	2,808.3	3,447.9
ICGV 15064	1,641.7	1,875.0		3,166.7	3,658.3	
ICGV 15060	1,700.0	1,550.0		4,475.0	2,033.3	
ICGV 15017	1,708.3	1,458.3		3,358.3	2,000.0	
ICGV 15008	1,750.0	1,650.0	1,395.8	2,800.0	2,308.3	2,916.7
ICGV 15039	1,766.7	1,858.3		2,933.3	1,200.0	
ICGV 15052	1,766.7	1,175.0		4,000.0	1,758.3	
ICGV 15035	1,783.3	1,466.7		2,508.3	1,416.7	
ICGV 15076	1,841.7	1,241.7	1,625.0	2,933.3	3,066.7	2,864.6
ICGV 15038	1,908.3	1,566.7		3,300.0	3,141.7	
ICGV 15074	1,958.3	1,425.0	1,218.8	3,575.0	1,916.7	3,416.7
ICGV 15083	2,025.0	1,416.7		2,933.3	1,858.3	
ICGV 15044	2,133.3	1,941.7		3,366.7	3,191.7	
ICGV 15065	2,400.0	1,691.7		4,050.0	1,991.7	

Overall, 18 parental lines have been included in development of a new generation of high oleate groundnut. The elite and released materials targeted include ICGV-SM 90704 (Nsinjiro), CG 7, ICGV-SM 08503, ICGV-SM 06729, ICGV-SM 01731, ICGV-SM 01711, Chalimbana, ICGV-SM 03517, ICGV-SM 99557, ICGV-SM 99551, ICGV-SM 07539, ICGV-SM 07502, ICGV-SM 0552, ICGV-SM 99568, ICGV-SM 99556, and ICGV-SM 09511. A second approach involves the use of SunOleic 95R donor material for elite-by-elite crosses. These material are at F3–F4 depending on whether they are short duration (Spanish and Valencia) and or medium genotypes and long duration (Virginia). A marker assisted selection approach will be used to support line conversion through backcross approach. The populations developed are at different stages of advancements, with the earliest target for release being 2022.

ICRISAT-Mali groundnut program also developed 10 new high oleic populations during the 2018 main rainy season using two high oleic parents (ICGV 15112, ICGV 16012) by crossing with three released (Fleur 11, ICGV-IS 13825, ICG 7878) and two dominant farmers (28-206, 47-10) varieties. The F1s are planted during 2019 offseason using irrigation. The resulting F2s will be planted during the 2019 main rainy season where leaf samples will be sent to high throughput phenotyping and genotyping (HTPG) platform at Intertek-Hyderabad, India for genotyping for MAS of F2 plants carrying the high oleic allele.

## Advances in Crop Improvement Research on Groundnut Allergens

Groundnut allergy is one of the serious food allergies which affect 1–2% of the world populations. Australia tops the list of the most highly affected countries (Sicherer and Sampson, 2014); other highly affected countries include USA (Sicherer et al., 2010; Nicolaou et al., 2010), Canada (Ben-Shoshan et al., 2009), Denmark and France (Morisset et al., 2002; Osterballe et al., 2005), and the United Kingdom (Grundy et al., 2002). Groundnut allergy is not only life threatening but also adversely affects life quality of groundnut-allergic individuals and their families. Currently, there is no vaccine to prevent groundnut allergy in sensitive individuals, medicine to alleviate the allergic effects, or methods to reduce allergen proteins in the groundnut products.

Groundnut seed contains 32 different types of storage proteins and 18 of them have allergen property (Pele, 2010). The allergens Ara h 1, Ara h 2, Ara h 3, and Ara h 6 are the major allergens with ability to cause life-threatening reactions such as anaphylaxis (Krause et al., 2010). Groundnut sequence analysis has identified several homologs of the allergen encoding genes viz. three for Ara h 1, one for Ara h 2, eight for Ara h 3, and two for Ara h 6 (Ratnaparkhe et al., 2014). The study by mining allergen genes in the reference genome of the diploid A genome (*A. duranensis*, accession PI475845) (Chen et al., 2016) and indirect transcriptome studies covering few seed development stages (Clevenger et al., 2016) provided inconclusive information on presence of allergen genes in the entire genome. Since the tetraploid genomes became available in 2019 (Bertioli et al., 2019; Chen et al., 2019; Zhuang et al., 2019), comprehensive genome and functional genomics studies are required for mining the genome-wide allergen genes so that crop improvement approaches can be deployed for developing groundnut varieties with low allergen contents. Most recently, ICRISAT-India developed monoclonal antibody based ELISA protocol that successfully screened diverse set of groundnut accessions identifying five major allergens Ara h 1, Ara h 2, Ara h 3, Ara h 6, and Ara h 8 as well as groundnut genotypes with low allergen contents (Pandey et al., 2019b; Pandey et al., 2019c). The threshold of allergen proteins differ significantly in the allergic population, for example a threshold of 100 µg of Ara h 1 is observed in some populations (Warner, 1999).

The recent studies by the U.S. FDA studies showed improved tolerance by introducing groundnut consumption during 4–10 months of age (https://www.fda.gov/food/cfsan-constituent-updates/fda-acknowledges-qualified-health-claim-linking-early-groundnut-introduction-and-reduced-risk). Another study has also demonstrated that beginning consumption of groundnut-containing foods in infancy (between 4 and 10 months of age) reduced the risk of developing groundnut allergy by 5 years of age by more than 80% (du Toit et al., 2018). An approved health claim by US FDA (https://www.fda.gov/food/cfsan-constituent-updates/fda-acknowledges-qualified-health-claim-linking-early-groundnut-introduction-and-reduced-risk) indicated that positive impact of early consumption of groundnuts may be one avenue to address potential groundnut allergies. For consideration of a qualified health claim regarding the relationship between the consumption of foods containing ground groundnuts and a reduced risk of developing groundnut allergy, the FDA found the scientific evidence appropriate and suggested to implementing agencies to provide clear information on the foods to avoid misleading consumers (https://www.fda.gov/media/107357/download). Further, FDA would monitor and evaluate for possible enforcement action situations where foods that bear the qualified health claim regarding reducing the risk of developing groundnut allergy that contain groundnuts in trivial amounts (https://www.fda.gov/media/107357/download). Nevertheless, if these efforts are more successful in increasing tolerance among kids in coming years, the groundnut lines with low allergen contents may provide an opportunity to be used in developing therapeutic product for vaccination or tolerance. Still a long way to go and much more efforts are needed in establishing the importance of low allergen protein containing groundnuts to be used as alternative and effective approach in fighting groundnut allergies.

## Exploiting the diploid and tetraploid groundnut genome sequences for crop improvement

The groundnut research community has witnessed rapid developments in this decade in the area of genomic resources which are critical for harnessing the potential of genomics for groundnut improvement (see Pandey et al., 2012; Varshney et al., 2013; Pandey et al., 2016; Pandey et al., 2017a; Pandey et al., 2019b; Pandey et al., 2019c). Availability of reference genome and high density genotyping assay are the most important milestones for understanding genome architecture, trait mapping, gene discovery, and molecular breeding (Varshney et al., 2013). The major genomic resources that have been developed in recent years include 1) reference genome of cultivated tetraploid (Bertioli et al., 2019; Chen et al., 2019; Zhuang et al., 2019); 2) reference genome of allotetraploid wild groundnut, Arachis *monticola* (Yin et al., 2018); 3) reference genomes of diploid progenitors of cultivated groundnut i.e., *A. duranensis* (Bertioli et al., 2016; Chen et al., 2016) and *A. ipaensis* (Bertioli et al., 2016; Lu et al., 2018); 4) “Axiom_Arachis” array, a high density genotyping assay with >58K highly informative SNPs (Pandey et al., 2017a); 5) gene expression atlas for cultivated tetraploid (Clevenger et al., 2016); 6) molecular/genetic markers (Pandey et al., 2016; Pandey et al., 2017a; Vishwakarma et al., 2017; Zhao et al., 2017; Lu et al., 2019; Pandey et al., 2019b; Pandey et al., 2019c); and 7) diverse genetic populations such as MAGIC and nested association mapping (NAM) populations to conduct high resolution genetic mapping and breeding (Pandey et al., 2017a; Pandey et al., 2017b; Pandey et al., 2017c); and 8) trait linked diagnostic markers for use in genomics-assisted breeding (GAB) (Pandey et al., 2017c). As a result, the next-generation sequencing based trait discovery (Pandey et al., 2017b) and sequence-based breeding (Varshney et al., 2019) will enhance breeding speed and precision for greater genetic gains.

So far, the reference genomes of diploid progenitors have been used for comparative genomics, structural and functional genomics, trait mapping, gene and marker discovery. Now the reference genome for the cultivated tetraploid groundnut (cultivar Tifrunner) has been reported, by the International Groundnut Genome Initiative (IPGI, https://groundnutbase.org/groundnut_genome), Fujian Agriculture and Forestry University^3^ and ICRISAT-India, and Crop Research Institute of the Guangdong Academy of Agricultural Sciences (GAAS), China (Chen et al., 2019). Since one genome is not enough, we should sequence complete GeneBank accessions of groundnut. In this context, ICRISAT has completed sequencing of Groundnut Reference Set which is a global diversity panel and further comparative structural genomics and association mapping is in progress. Such efforts are likely to be increased in groundnut in the coming years.

## Delivering Advanced Genetics to Smallholder Farmers to Unlock Groundnut Production

Once superior groundnut varieties with improved nutritional quality (high oleic acid, low allergenic properties, low aflatoxin producing) are developed and released, sustained multi-sectoral participatory efforts of groundnut scientists, nutritionists, public health experts, socio-economists, nongovernmental organizations (NGOs), policy makers from the governments, and civil society champions is needed to develop functional delivery models that improve effectiveness of various production-to-consumption value chains (Ojiewo et al., 2019).

Market-oriented and/or export-led commercial production is a necessity for sustainable legume value chain (Rubyogo et al., 2019). This is still lacking in many developing countries, where a significant proportion of groundnut production is mostly done by small-scale farmers under rainfed conditions for subsistence (Ojiewo et al., 2018a). McGuire and Sperling (2016), in a large sample of 2,592 smallholder farmers in six countries found that only 7% of legume seed came from the formal or semi-formal (agro-dealers, government aid, NGOs, community seed groups) sectors. Therefore, 93% came from informal sources. Of this, 64% is purchased from local markets, mostly from grain aggregators/grocery stores. Similarly in North-eastern Nigeria, only 9% of farmers purchase seeds from seed companies, while 22% purchase from local market/grain aggregators (Ajeigbe et al., 2018). There is, therefore, evidence that farmers do buy legume seed. However, for various reasons they do not buy legume seed from the formal outlets.

Preliminary results from a study being conducted in Uganda on Gender Integration in Seed Systems indicate that only 2% of farmers save their own seed and plant the next season. Most smallholder legume farmers produce and consume or sell all their produce before the planting season to meet their basic needs and are therefore compelled to purchase seed during planting season. A few farmers, with alternative sources of income, manage to save grain from their harvest for better prices during shortages just before planting season and this is often the source bought by grain aggregators and later sold to other farmers. The greatest concern is the poor quality of the “seed” obtained from such market sources. Many times, the seed has to be sorted with significant sorting loss and suffers poor germination, vigor, and crop establishment as well as potential for seed borne diseases.

Farmers would potentially change their behavior to source seed from high quality sources if they are made aware of these losses and if access to high quality seed at reasonable prices is facilitated/arranged, and stable prime price market is guaranteed. From the same study, it was noted that farmers do not understand the language of “certified seed,” but are actually interested in and willing to pay for high quality groundnut seed. Their sense of quality is in the color from inherent knowledge of an old variety called ‘Red Beauty'. They associate any red variety with this old variety and assume that all red colored groundnut is improved variety and seeds of the same are of high quality. Farmers here suggest that simpler language such as “super seed” would convey the language of quality better than “certified seed”.

Variety release and adoption figures summarized by the CGIAR DIIVA (Diffusion and Impact of Improved Varieties in Africa) project data on selected crops in sub-Saharan Africa (http://www.asti.cgiar.org/diiva), suggest that many of the improved groundnut varieties are not adopted and produced by farmers. This leaves a number of unanswered questions about the productivity and profitability/value of new crop varieties: Are the varieties superior/good enough? Do we have robust data on superiority (productivity/profitability) of new varieties to convince the private sector to commercialize them? Are value chain actors aware of them? Is the seed system ready to respond to demand? It is important to establish a product advancement criteria and process to prioritize varieties for commercialization, backed by extensive on-farm testing systems and robust demonstration trial data, helping to make confident conclusions and recommendations for variety turnover by public and private sector seed enterprises. This process works to make research and development more business oriented by focusing on the decision-making criteria of markets and advancing a defined selection of varieties.

Besides, innovative and transformative models for accessing, multiplying, and disseminating public-bred varieties should be developed and promoted by scaling up seed enterprises. Innovative models of early generation (breeder and foundation) and certified seed production should be tested through demand-led public and private partnerships. Quality seed of improved varieties of groundnut is difficult to access in many countries due to bottlenecks in the early generation seed (EGS) value chain. This is due to a number of factors related to perceived marginal economic value of quality seed. Some of the major factors that are important for a successful seed value chain include grain demand for varieties produced with quality seed, national and regional policy environment, quality assurance mechanisms, capacity and resources across the seed value chain, organization and implementation of quality assurance mechanisms, as well as quality of physical infrastructure. For example, with increase in demand from food industries for genetically pure high oleic groundnut for private seed companies are expected to play a crucial role in responding to this demand and in ensuring a more organized sustainable seed supply chain. Seed farmers or seed entrepreneurs will be the direct beneficiaries of the system as their help will be needed to meet the high demand. Thus, the development and release of high oleic acid groundnuts creates a demand-pull thus benefitting all the groundnut value chain actors, which include farmers, and food and oil industries, along with providing healthy alternatives for consumers.

Seed Revolving Fund (SRF) is a model developed and successfully implemented by ICRISAT and partners in Malawi to address limited production and supply of groundnut EGS (Siambi et al., 2015). The model involves public and private partners at each stage of the seed value chain where breeder seed is produced and supplied by the public breeding institution. Farmer seed producer groups are trained in quality seed production, and contracted by the SRF to produce foundation seeds, at agreed buy-back prices. Individual larger scale farmers are contracted to multiply seed for the SRF, especially if they have irrigation facilities that secure production even in drought years. It is important for entrepreneurs to purchase breeder seed instead of the SRF providing seed and deducting from the sales. Foundation seed is then sold to local seed ventures for multiplication into certified seeds. The companies produce and sell certified seed through agro-dealers. Proceeds of the sales realized through the SRF are ploughed back to cover the operational costs such as staff, inspection and certification, warehouse, seed packaging and transport, and this enables the fund to engage more entrepreneurs every year. The success of the SRF model requires strict standard operating procedures to ensure good quality seed and also avoid conflict of interest by staff. Another important requirement is that the proceeds from the sales must “revolve” to enable the unit to make further investments and carry out all the necessary operations in a timely manner. This may involve consultation with governments to set up financial management structures that provide an easier accountability process. Seed quality is assured through a strategic partnership with the government's Seed Services Unit. It is also necessary to link up with grain and commodity markets, especially processors, to ensure sustained demand for grain, which then pulls the seed.

## Conclusion and Future Prospects

Investment in conventional breeding to develop groundnut varieties with low to zero aflatoxin contamination in groundnut is slowly making progress especially over the last decade. Key developments include exploiting resistance to the pathogen *A. flavus* and *A. parasiticus*, focusing on partial resistance during seed colonization, pre-harvest aflatoxin contamination in the field, and reduction of aflatoxin production. Further, transgenic approaches using plant defensins and host-induced gene silencing hold promise for elimination of aflatoxin production in nuts at both pre-harvest and post-harvest stages. While genotypes with very low allergen proteins have been identified, development of genomics assisted breeding will hasten deployment of the low allergenic trait groundnut varieties. The current effort to use genomic assisted breeding for development of high oleate and low linoleic and palmitic acids groundnut, have potential to unlock competitiveness of groundnut providing opportunity from farm-to-fork. The seed industry could benefit from the increasing demand for propriety groundnut, such as high oleate genotypes, to develop a market oriented systems. Leveraging on such a systems, the regular groundnut and other legumes could benefit, further strengthening resilience of faring communities. Taken together, the increasing demand from food industries for high oleate groundnut, with low allergenic and aflatoxin properties, an organized seed sector leveraging on advances in science, the third industrial revolution underpinned by information and communication technology (ICT), improvements in finance inclusivity and policy groundnut provides a good model crop to meet production to consumption and income benefits to millions of households who depend on the crop.

## Author Contributions

CO wrote the first draft and incorporated all inputs from the coauthors and editors. HD, PO, JM, HA, WG-W, PB-M, PJ, MP, and RKV contributed to the section on *Advances In Crop Improvement to Mitigate Groundnut Aflatoxin Contamination*. PB-M further contributed to the sub-section on *Aflatoxin Mitigation in Groundnut Using Host‐Induced Gene Silencing and Transgenic Approaches: Technology and Translation*. PJ, MV, TR, KD, SB, AR, NM, RPV, and MK contributed the section on *High Oleic Groundnut Varieties for Food and Nutrition Security*. MP and RKV contributed the sections on *Advances in Crop Improvement Research on Groundnut Allergens* and *Exploiting the Diploid and Tetraploid Groundnut Genome Sequences for Crop Improvement*. CO, EN-M, GM, PO and EA contributed to the section on *Delivering Advanced Genetics to Smallholder Farmers to Unlock Groundnut Production*.

## Funding

The funding support for this study was received from the Bill and Melinda Gates Foundation (fund number OPP1114827), National Mission on Oilseeds and Oilpalm (NMOOP), Department of Agriculture and Co-operation (DoAC), Ministry of Agriculture, Government of India, Biotechnology (DBT) of Government of India; National Agricultural Science Fund (NASF) of Indian Council of Agricultural Research and MARS Wrigley, USA. The work reported in this article was undertaken as a part of the CGIAR Research Program on Grain Legumes and Dryland Cereals (GLDC). ICRISAT is a member of the CGIAR.

## Conflict of Interest

The authors declare that the research was conducted in the absence of any commercial or financial relationships that could be construed as a potential conflict of interest.

## Footnotes

1
https://www.nature.com/articles/d41586-018-01642-w

2
https://www.nature.com/articles/d41586-019-01643-3

3
http://groundnutgr.fafu.edu.cn
